# 1331. Seasonality of Common Human Coronaviruses in the United States, 2014-2021

**DOI:** 10.1093/ofid/ofab466.1523

**Published:** 2021-12-04

**Authors:** Melisa Shah, Amber K Haynes, Rebecca M Dahl, Krista Kniss, Benjamin Silk, Marie E Killerby

**Affiliations:** 1 Centers for Disease Control and Prevention, Atlanta, Georgia; 2 NCIRD/DVD, Atlanta, GA, Georgia; 3 CDC, Atlanta, Georgia; 4 Division of Viral Diseases, CDC, Atlanta, Georgia

## Abstract

**Background:**

The four common human coronavirus (HCoV) types, including two alpha (NL63 and 229E) and two beta (HKU1 and OC43) coronaviruses, generally cause mild, upper respiratory illness. Common HCoV seroprevalence increases rapidly during the first five years of life and remains high throughout adulthood. HCoVs are known to have seasonal patterns, with variation in predominant types each year, but more defined measures of seasonality are needed.

**Methods:**

We describe laboratory detection, percent positivity, and seasonality of the four common HCoVs during July 2014 to May 2021 in the United States reported to the National Respiratory and Enteric Virus Surveillance System (NREVSS). We also describe age, sex, and co-detection with other respiratory viruses for a subset of specimens available through the Public Health Laboratory Interoperability Project (PHLIP). We used a method previously validated for respiratory syncytial virus, characterized by a centered 5-week moving average and normalization to peak, to define seasonal inflections, including season onset, peak, and offset.

**Results:**

Any HCoV type was detected in 96,336 (3.4%) of 2,487,736 specimens. Predominant common HCoV types fluctuated by surveillance year (Figure 1) and were generally consistent across geographic regions. In a subset of 4,576 specimens with a common HCoV detection, those with type 229E had a higher median age compared to other HCoV types (30.8 versus 24.8 years, p< 0.001), but there were no differences by sex. Influenza was the most commonly co-detected virus. In the last six complete HCoV seasons, onsets ranged from October to November, peaks from January to February, and offsets from April to June; >95% of all HCoV detections occurred within these ranges. The 2020-2021 common HCoV season onset, dominated by types NL63 and OC43, was delayed by approximately two months compared to prior seasons.

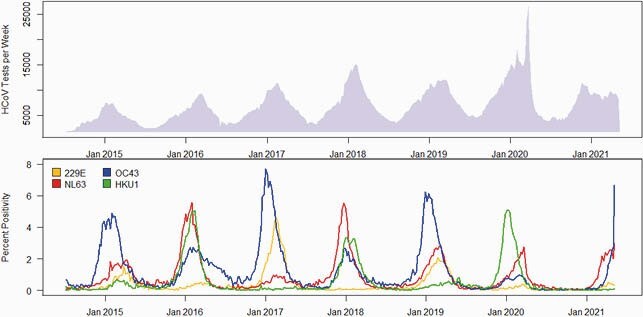

Figure 1. The top panel represents total specimens tested and the bottom panel shows percent positivity of the four common human coronavirus (HCoV) types by week starting July 5, 2014 through May 8, 2021. Data are from the National Respiratory and Enteric Virus Surveillance System (NREVSS).

**Conclusion:**

Common HCoVs demonstrate relatively consistent seasonal patterns. The delayed onset of the 2020-2021 season may be attributable to mitigation measures implemented across the US including masking, improved hand hygiene, and social distancing. Better defining HCoV seasonality can inform clinical preparedness and testing practices and may provide insights into the behavior of emerging coronaviruses.

**Disclosures:**

**All Authors**: No reported disclosures

